# A Murine Model of Variant Late Infantile Ceroid Lipofuscinosis Recapitulates Behavioral and Pathological Phenotypes of Human Disease

**DOI:** 10.1371/journal.pone.0078694

**Published:** 2013-11-01

**Authors:** Jeremy P. Morgan, Helen Magee, Andrew Wong, Tarah Nelson, Bettina Koch, Jonathan D. Cooper, Jill M. Weimer

**Affiliations:** 1 Division of Basic Biomedical Sciences. Sanford School of Medicine at the University of South Dakota, Vermillion, South Dakota, United States of America; 2 Department of Pediatrics, Sanford School of Medicine at the University of South Dakota, Sioux Falls, South Dakota, United States of America; 3 Children's Health Research Center, Sanford Research/USD, Sioux Falls, South Dakota, United States of America; 4 James Black Centre at the Institute of Psychiatry, King's College, London, United Kingdom; 5 Department of Neuroscience, King's College, London, United Kingdom; 6 Department of Biochemistry, Children's Hospital, University Medical Center Hamburg-Eppendorf, Hamburg, Germany; University of Edinburgh, United Kingdom

## Abstract

Neuronal ceroid lipofuscinoses (NCLs; also known collectively as Batten Disease) are a family of autosomal recessive lysosomal storage disorders. Mutations in as many as 13 genes give rise to ∼10 variants of NCL, all with overlapping clinical symptomatology including visual impairment, motor and cognitive dysfunction, seizures, and premature death. Mutations in *CLN6* result in both a variant late infantile onset neuronal ceroid lipofuscinosis (vLINCL) as well as an adult-onset form of the disease called Type A Kufs. CLN6 is a non-glycosylated membrane protein of unknown function localized to the endoplasmic reticulum (ER). In this study, we perform a detailed characterization of a naturally occurring Cln6 mutant (*Cln6^nclf^)* mouse line to validate its utility for translational research. We demonstrate that this *Cln6^nclf^* mutation leads to deficits in motor coordination, vision, memory, and learning. Pathologically, we demonstrate loss of neurons within specific subregions and lamina of the cortex that correlate to behavioral phenotypes. As in other NCL models, this model displays selective loss of GABAergic interneuron sub-populations in the cortex and the hippocampus with profound, early-onset glial activation. Finally, we demonstrate a novel deficit in memory and learning, including a dramatic reduction in dendritic spine density in the cerebral cortex, which suggests a reduction in synaptic strength following disruption in CLN6. Together, these findings highlight the behavioral and pathological similarities between the *Cln6^nclf^* mouse model and human NCL patients, validating this model as a reliable format for screening potential therapeutics.

## Introduction

The neuronal ceroid lipofuscinoses (NCLs) are a family of fatal lysosomal storage diseases composed of at least 10 disease variants (Reviewed in [1], [2], [3]). These diseases are classically characterized by accumulation of autofluorescent storage material within cells of the brain and other tissues and mutations in as many as 13 genes have been reported to cause NCLs (Reviewed in [3], [4], [5]; see http://www.ucl.ac.uk/ncl/mutation.shtml). Although genetically distinct, this family of disorders shares overlapping disease symptomatology, including early onset visual deterioration, declining motor coordination, frequent seizures, mental deterioration, and premature death (Reviewed in [6], [7]).

Mutations in *CLN6* result in both a variant late infantile NCL (vLINCL) and adult onset type A Kufs disease (MIM#601780, www.omim.org; [8], [9], [10], [11], Reviewed in [12]). *CLN6* is an approximately 22.7 kb gene located on chromosome 15q23 [11]. Its 7 exons code for a 2.4 kb mRNA transcript which results in a 311 amino acid protein with 7 transmembrane domains [11], [13]. CLN6 contains two ER retention signals, one found on the N-terminal cytoplasmic domain, and the other on the C-terminal luminal domain [14], [15]. This non-glycosylated protein can homodimerize within the ER, although its precise function there remains unknown [14]. The most common mutation in CLN6, which leads to vLINCL, results from the insertion of an additional cytosine at base pair 307 in exon 4, leading to a frameshift and premature stop codon. vLINCL disease onset occurs between 18 months and eight years of age, with symptoms of motor delay, vision loss, dystharthia, and ataxia followed by premature death during the second decade of life [16], [17].

*In vivo* NCL models have also been invaluable in dissecting disease pathologies. For vLINCL, these models include the New Zealand South Hampshire sheep (OCLN6) and the Merino sheep [18], [19], [20], a mouse model (*Cln6^nclf^*) [21], and a dog model [22]. Pathologically, diseased sheep mirror human patients with severe cortical atrophy, widespread glial activation, and accumulation of autofluorescent storage material [18], [20], [23], [24], [25], [26], as well as presenting with motor dysfunction, vision loss and seizures [27]. The *Cln6^nclf^* mouse model, identified at The Jackson Laboratory (Bar Harbor, ME), develops hind-limb paralysis around 8 months and dies prematurely around 1 year [21]. These mice displayed retinal degeneration as early as 6 months of age and intracellular inclusions were detected as early as 11 days of age, demonstrating the early onset nature of the disease. Similar to the ovine model, reactive hypertrophic astrocytes are visible in the cerebral cortex, hippocampus, thalamus, and brain stem of *Cln6^nclf^* mice by 6 months of age [13],[21].

In addition to providing valuable insight into disease pathogenesis, these *in vivo* models have also started to provide clues into CLN6's function and in defining what role protein disruption may plays in disease. For instance, cDNA microarray analysis of CLN6 deficient fibroblasts has suggested involvement of CLN6 in extracellular matrix modulation, signal transduction pathways, apoptosis, and immune/inflammatory response pathways [28]. Protein-protein interaction studies have demonstrated binding of CLN6 to the collapsin response mediator protein-2 (CRMP-2), suggesting a role of CLN6 in axonal transport, elongation or maintenance [29]. Several recent studies in both mouse and sheep models have demonstrated that loss of CLN6 leads to a disruption in synaptic function and/or levels of essential synaptic proteins [30], [31]. Changes in cholesterol dynamics in CLN6 deficient cells have hinted a role of this protein in regulating structure and function of caveolae and lipid rafts, as well as protein sorting mechanisms [28]. Additionally, loss of CLN6 leads to disruption of the autophagy-lysosome degradation pathway [32] and has been linked to defects in biometals (such as zinc, copper manganese, and cobalt) homeostasis- both pathologies similar to other neurodegenerative diseases [30], [33]. Studying these animal models has also provided valuable insight into the composition of the storage material in CLN6 defective cells – demonstrating the presence of subunit C of the mitochondrial ATP synthase ([19], reviewed in [24], [34]).

In this study, we validate that *Cln6^nclf^* mice are an accurate and reliable model of the human vLINCL, displaying many of the same pathological changes as patients, including significant cortical atrophy, massive accumulation of autofluorescent storage material, microglial and astrocytic activation, and interneuron loss within the hippocampus and cerebral cortex - all hallmarks of NCLs. Within the motor cortex, we observed a decrease in dendritic spine density on excitatory glutamatergic pyramidal neurons. We demonstrated that they develop motor and cognitive dysfunction as well as profound visual impairment. Taken together, these findings demonstrate the significant value of *Cln6^nclf^* mice as a small mammalian model for vLINCL and future drug screening.

## Methods

### 1. Ethics statement/Animals

Animal protocols were approved by the Sanford Research/USD Institutional Animal Care and Use Committee (USDA License 46-R-0009) with all procedures carried out in strict accordance with National Institutes of Health guidelines and the Sanford Research/USD Institutional Animal Care and Use Committee guidelines. Wild-type (WT) and homozygous Cln6-mutant mice (*Cln6^nclf^*) on C57BL/6J backgrounds were utilized for all studies and were housed under identical conditions. For the studies presented here, only male mice were used. Body weights of mice were measured and analyzed by two-way ANOVA with Bonferroni's post-hoc test. An allelic discrimination assay was developed for genotyping *Cln6^nclf^* DNA from tissue samples prepared using the Wizard SV Genomic DNA Purification System (Promega, Madison, WI) and normalized to 5 ng/μl prior to mixing with 2X ABsolute Blue qPCR mix containing Rox dye at 60 nM, 20X *Cln6^nclf^* custom TaqMan SNP genotyping assay (Applied Biosystems, Carlsbad, CA), and DNase free water. Primer/Probes in the custom *Cln6^nclf^* assay were: Forward: CCCTCATTCTTCACCTCAGCTTATT, Reverse: GATGAAAGTGATGATGCTGACATAGACT, WT/FAM reporter: CGGTCCCCCCGAACG, and *Cln6^nclf^*/VIC reporter: CGGTCCCCCCCGAACG. The qPCR cycle was run at 95°C for 15 min followed by 40 cycles of 95°C for 15 seconds, and 60°C for 60 seconds.

### 2. Behavioral assessment

For all behavioral testing, animals were habituated and tested at the same time each day and housed in a room with a 12 hour light–dark cycle between testing.

#### Rotarod testing

An accelerating rotarod (AccuScan instruments) was used to measure motor coordination over time in *Cln6^nclf^* and control mice as previously described [35], [36], [37]. In brief, *Cln6^nclf^* and WT mice (*n* = 7-10) were tested at day 14, 28, 90, and 270 by placing them on the rotarod starting at 0 rotations per minute (RPM) and accelerating to 30 RPM over a period of 240 seconds. Three consecutive runs for each test trial were averaged and the latency to fall during the testing period was calculated. The same cohorts of mice were tracked over time and data were analyzed by two-way ANOVA with Bonferroni's post-hoc test.

#### Visual cliff

The visual cliff was used to assess visual performance. The apparatus consists of a wooden box with a plexiglass bottom that is placed halfway over the edge of a table to create the illusion of a cliff, as previously described [38], [39]. A lamp was lit from underneath the apparatus to emphasize the visual cliff. Mice were placed in the apparatus, and the time spent over the visual cliff in a single fifteen minute trial was measured. Data were analyzed by Student's t-test and presented as mean ±S.E.M.

#### Pole climb

A standard pole climb test was utilized to assay motor balance and coordination using standard climb down and turn down assays [40], [41]. Climb down: Mice were placed face-down at the top of a threaded metal rod (diameter 1.27 cm, height 60 cm). The time for the mice to climb to the bottom of the pole and place all 4 feet on the ground was measured. Five trials were performed, with a maximum time of 60 seconds. Turn down: Mice were placed face-up at the top of a threaded metal rod (diameter 1.27 cm, height 60 cm). The time for the mice to turn downward was measured. Five trials were performed, each with a maximum time of 60 seconds. Data were analyzed by Student's t-test.

#### Open Field Motor Performance

Automated open field locomotor activity was assayed using a chamber equipped with infrared photobeams (Stoetling ANY-maze, Wood Dale, IL) to measure total distance traveled as previously described [42]. In brief, 6-month old mice were habituated to the locomotor activity chamber for one 15-min session. On the following day, locomotor testing was performed identical to the pre-testing habituation for 15 min. Photobeam breaks were recorded every 50 ms for 30 min for horizontal, vertical and ambulatory movements. Data were analyzed by Student's t-test.

#### Radial arm

A standard radial arm maze was used to examine learning and memory [43] using a video tracking and analysis system (Stoetling ANY-maze, Wood Dale, IL). Mice were food-deprived 24 hours prior to the start of the radial arm maze, followed by continuous food restriction throughout the test period. The mice received a habituation period in which they were placed in the radial arm maze apparatus for 5 minutes a day for 3 consecutive days, with a sucrose pellet available at the end of each of the 8 arms. A training period was then performed for 17 days, in which the mice were placed in the radial arm apparatus for 8 five-minute trials per training day, and sucrose pellets were placed at the end of 3 arms (the trained sequence). To evaluate memory, mice were placed in the apparatus for 8 five-minute trials and sucrose pellets were placed in the same three arms as for the training period. The latency to complete the trained sequence by reaching the end of the 3 arms at least once, and the number of errors performed (the number of entries into arms that were not part of the trained sequence) were measured using ANY-maze software (Stoelting Co., Wood Dale, IL). A learning test was performed on the subsequent day, in which the ability of the mice to learn a new 3-arm sequence was evaluated in 8 five-minute trials. Sucrose pellets were placed at the ends of three arms different from the originally trained sequence, with the latency to complete the new sequence and the number of errors measured. Data were analyzed by Student's t-test and presented as mean ±S.E.M.

### 3. Histological and stereological characterization

#### Histological processing

For histological analysis of the retina, eyes were removed from euthanized WT and mutant mice at postnatal day 0 (P0), P90, and P240 and fixed in paraformaldehyde. The lenses of the eyes were removed, samples were dehydrated in graded ethanol, cleared in xylenes, embedded in paraffin, sectioned at 6 µm and stained with Hematoxylin and Eosin as described previously [44].

For histological analysis of the brains, age-matched WT and mutant mice were deeply anaesthetized with sodium pentobarbital (100 mg/kg) and transcardially perfused with a vascular rinse (0.8% NaCl in 100 mM NaHPO_4_) followed by fixation with 4% paraformaldehyde as previously described [45]. Brains were post-fixed overnight, cryoprotected in 30% sucrose, and 40 µm frozen coronal sections were collected in cryoprotectant solution [Tris-buffered saline (TBS)/30% ethylene glycol/15% sucrose/0.05% sodium azide].

#### Nissl staining

To visualize neuronal morphology, every 6 th section was mounted onto gelatin-chrome alum slides, air dried and incubated at 60°C in a solution of 0.05% cresyl fast violet and 0.05% acetic acid, differentiated through a graded series of ethanol before clearing in xylene, and coverslipped.

#### Histological Analysis

For immunological examination, *Cln6^nclf^* and WT sections were processed as previously described [44], [46]. To survey a variety of phenotypic markers normally expressed in subpopulations of cortical and hippocampal cells, adjacent one-in-six series of free floating frozen sections were labeled immunohistochemically as previously described [45], [47]. The following antibodies were used: anti-parvalbumin; anti-calretinin; anti-calbindin (all from Swant, Bellinzona, Switzerland), anti-somatostatin (Peninsula Laboratories, San Carlos, CA), anti-CD68 (Serotec, Oxford, United Kingdom), and anti-GFAP (DAKO, Cambridge, UK). Samples were either incubated in appropriate Alexa-Fluor secondary antibodies (Molecular Probes, Carlsbad, CA) and DAPI nuclear stain or conjugated with a Avidin: Biotin enzyme Complex (Vector Labs, Burlingame, CA) and immunoreactivity visualized by incubation in 0.05% 3,3′-Diaminobenzidine (DAB) (Sigma, Dorset, UK). Data were analyzed by one-way ANOVA with Bonferroni's post-hoc test and presented as mean ±S.E.M.

#### Storage Material

Visualization of autofluorescent storage material was performed by capturing confocal images from various regions of the CNS as described previously [48], [49]. All images were captured using the 543 nm laser and 40X objective on a Zeiss Pascal LSM 5 microscope (Carl Zeiss Ltd, Welwyn Garden City, UK), maintaining a consistent relative relationship between amplitude offset and detector gain between samples.

#### Stereology

Unbiased Cavalieri estimates of volume and cell numbers of the cortex and hippocampus were performed as previously described [35],[36],[45],[50]. In brief, Nissl stained sections of age-matched 5 month and 9 month mice were analyzed using StereoInvestigator software (Microbrightfield Inc., Williston, VT) on a Zeiss, Axioskop2 MOT microscope (Carl Zeiss Ltd, Welwyn Garden City, UK) linked to a DAGE-MTI CCD-100 camera (DAGE-MTI Inc., Michigan City, IN, USA), with no prior knowledge of genotype [51]. A sampling grid (250 µm^2^ cortex and 200 µm^2^ hippocampus) was superimposed over sections and the number of points covering the relevant areas were counted using a 5X objective. Regional volumes were collected from various cortical subregions and the hippocampus, and the mean volume of each region was calculated and analyzed by Student's t-test and presented as mean ±S.E.M. Cortical thickness measurements were collected from Nissl stained sections of age-matched 1, 4, 6, and 11 month mice in motor (M1), somatosensory (S1BF), and lateral entorhinal (LEnt) regions of cerebral cortex, as previously described [45]. Measures of laminar thickness were performed in both primary M1 and primary S1BF cortex for individual laminae I, IV (S1BF only), V, and VI, while a combined measurement was taken for laminae II and III, as previously described [45], [47]. Data were analyzed by Student's t-test and presented as mean ±S.E.M. Image threshold analysis measurements of 20X micrograph sections immunostained with astrocyte and microglial specific antibodies [GFAP (Dako, Cambridge, UK) and CD68 (AbD Serotec, Kidlington, UK)] were made at various time points (GFAP at 1, 4, 6 and 11 months; CD68 at 1, 4, and 6 months); data were analyzed by Student's t-test and presented as mean percent immunoreactivity as previously described [45], [50], [52].

#### Golgi-Cox staining and dendritic spine analysis

Brains of age -matched 2 month old WT and mutant mice were immersed into Golgi-Cox staining solution (7.14 M potassium dichromate, 7.14 M mercuric chloride, and 5.7 M potassium chromate) for 2 weeks, followed by 7 days of incubation in 30% sucrose and sectioning at 75 µm. Sections were reacted with 50% ammonia solution and 1% sodium thiosulfate. Glutamatergic pyramidal neurons from layers III-VI of the medial (M1/S1BF) cortex were analyzed for spine density. Dendrites were analyzed if the length of the dendrite extended 75 µm past the soma. Images were captured on the Nikon Eclipse 90i (Nikon Instruments Inc., Melville, NY) microscope and magnified by 60X (plus 2X optical zoom) with Z-stacks captured at approximately 0.6 µm thickness. Enhanced depth focus (EDF) images were created and ImageJ Mosaic was used to tile EDF images. Spine density measurements were performed by counting visible spines (ImageJ Cell Count), and dividing by length of the dendrite. Area of spine heads and spine lengths were quantified using Nikon Elements tracing software (Nikon Instruments Inc., Melville, NY). Data were analyzed by Student's t-test and presented as mean spine density (a minimum of 40 neurons were counted, three animals were analyzed per genotype).

## Results

### Common pathological and behavioral NCL characteristics observed in the *Cln6^nclf^* mice

**Figure 1:**
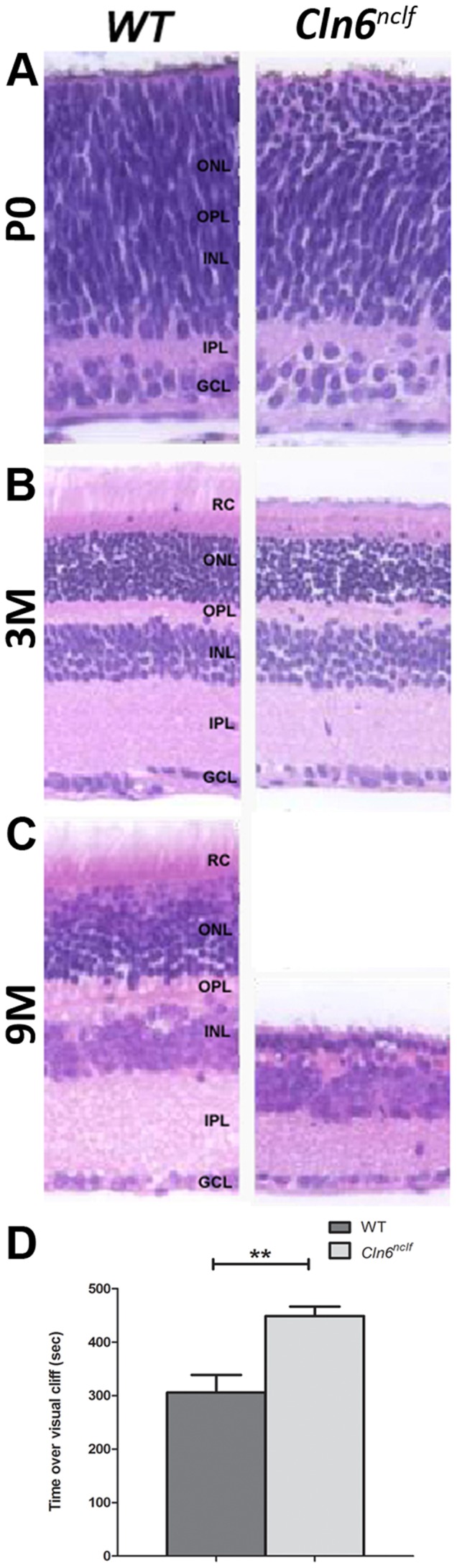
Retinal degeneration and vision loss in the *Cln6^nclf^* mouse. Cell loss and structural degenerative changes occur in the retina of *Cln6^nclf^* mice. (**A**) Comparison of gross morphological changes over time in retina of *Cln6^nclf^* mice and their respective age-matched WT controls was done to determine mechanism of degeneration. (**B**) Micrographs (4X) show a section of one retinal hemisphere. (**C**) At P0 all layers are present and of equal thickness. By 3 months of age the *Cln6^nclf^* retina has begun to narrow and shows a distinct loss of the rods and cones while the overall cytoarchitecture remains intact. By 9 months the rods and cones are nearly absent and the outer plexiform layer is virtually nonexistent with the merging of a much thinned outer and inner nuclear layers. Additionally, there is a distinct narrowing of the inner plexiform layer. [RC-Rode/Cone layer; ONL-Outer nuclear layer; OPL-Outer plexiform later; INL-Inner nuclear layer; IPL-Inner plexiform layer; GCL-Ganglion cell layer]. (D) At 8 months of age, *Cln6^nclf^* mice displayed a significant reduction in visual acuity in a visual cliff assay. Mutant mice were unable to distinguish between a “safe” region of the visual cliff box versus the “unsafe” cliffed portion, spending equal time between the two regions. [Mean (in seconds) +/- SEM, *n* = 6–9 mice per group (**p≤0.01)].

Retinal atrophy and progressive vision loss is a pathological hallmark of vLINCL patients (reviewed in [6], [53], [54]). *Cln6^nclf^* mice show a similar progressive loss of cells within the retina. At postnatal day zero (P0), all layers are present and of equal thickness (Fig. 1 A). By 3 months the *Cln6^nclf^* retina has begun to narrow and shows a distinct loss in the photoreceptor layer with both inner and outer nuclear layers showing disorganization (Fig. 1 B). By 9 months there is massive degeneration, with the rods and cones nearly absent, the outer plexiform layer (OPL) virtually nonexistent, and a merging of the outer (ONL) and inner nuclear layers (INL). There is also a distinct narrowing of the inner plexiform layer (IPL) demonstrating that by 8 months of life, the retina of the *Cln6^nclf^* is very severely deteriorated (Fig. 1 C). To assay visual performance and acuity, mice were analyzed in a visual cliff paradigm [38], [39]. 8 month old *Cln6^nclf^* mice were unable to discriminate between the safe zone and crossing over the cliff zone, spending equal amounts of time within each compartment (Fig 1 D), demonstrating a significant loss in vision at this time point.

The NCLs are classified based on the accumulation of autofluorescent storage material in both the CNS and peripheral tissues. *Cln6^nclf^* mice also display this hallmark with storage material detected in the cortex [21]. We observed a similar pattern of accumulation – where at 5 months intracellular accumulation of storage material was detected in *Cln6^nclf^* mice, which progressed significantly by 9 months (Fig. S1), mirroring the progressive accumulation seen in human patients.

**Figure 2:**
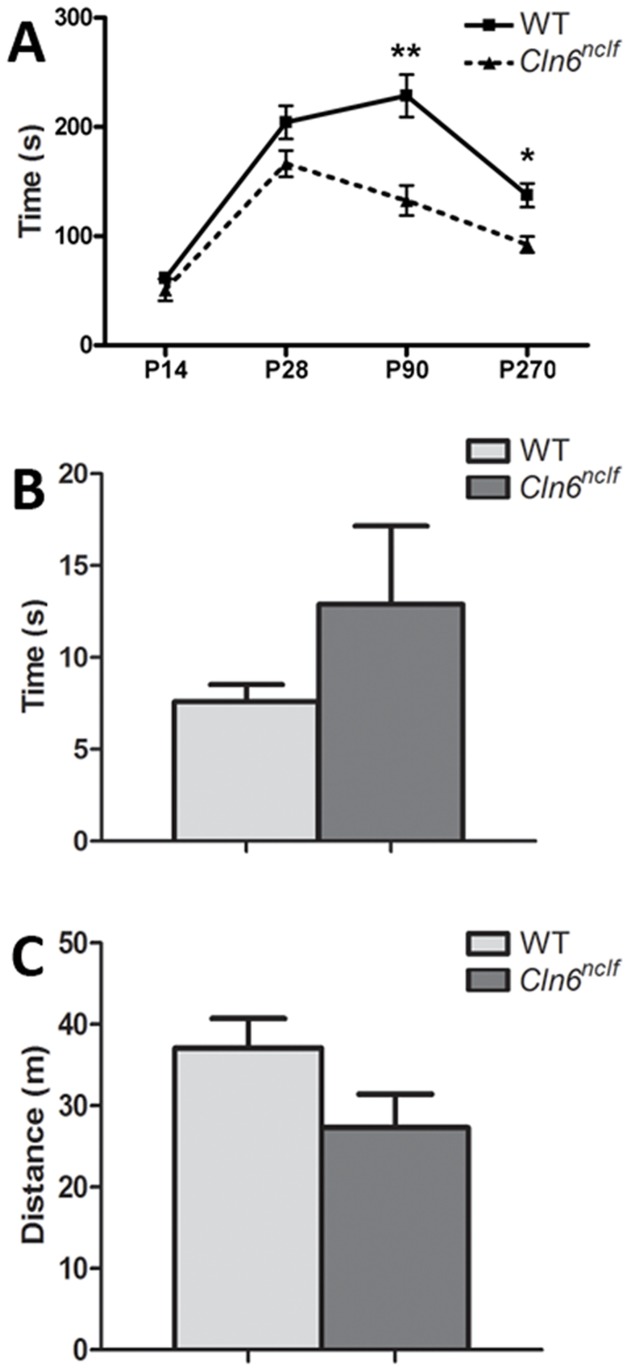
Decreased motor coordination deficits in *Cln6^nclf^* mice. **(A)** Rotarod testing was performed on postnatal day 14, 28, 90, and 270 old WT and *Cln6^nclf^* mice. Data are plotted as average latency to fall from the rotating rod during a 240 second trial period (3 trials per mouse per time point). *Cln6^nclf^* mice had a significant reduction in their ability to remain on the rod as it accelerated, starting at P90 and continuing at P270. **(B–C)** At six months of age, no difference was noted in additional motor performance measures including the time required to descend in a pole climb test **(B)** or the mean distance traveled (in meters) over a 15-min test period in an open field activity test **(C)**. [Mean +/− SEM, *n* = 6–9 mice per group (**p≤0.01, ***p≤0.0001)].

NCL patients often display severe motor dysfunction with symptoms presenting in vLINCL patients at approximately 18 months of age and including progressive motor delay, dysarthria, and ataxia ([10], [55], Reviewed in [56]). Here, we examine motor performance in *Cln6^nclf^* mice using several motor performance assays. First, rotarod testing was used to measure the ability of the mice to maintain their balance on a spinning rod. At P14 and P28 there is no difference in the latency to fall from the rod (Fig. 2 A). However, by 3 months there is a dramatic reduction in the ability of the mice to remain on the rod, indicating motor coordination and balance deficits. These deficits worsen over time, demonstrating early onset and progressive motor decline in this animal model of vLINCL (Fig. 2 A). General motor activity tests were performed using a standard pole climb and open field activity tests (Fig. 2 C–D), although no differences were noted at 6 months of age (pole climb, p = 0.22; open field, p = 0.1), suggesting these deficits are specific to balance and coordination.

**Figure 3:**
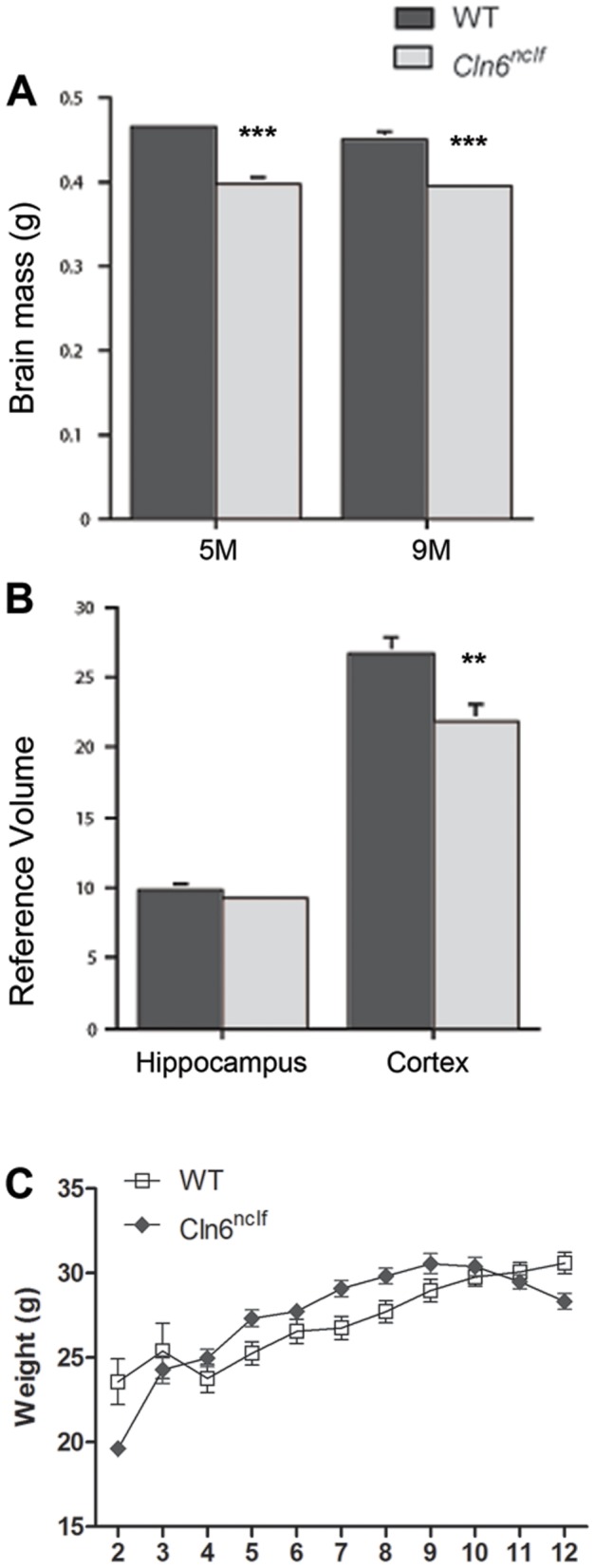
A reduction in brain mass and cortical volume seen in the adult *Cln6^nclf^* mouse. Brain mass was assessed in the *Cln6^nclf^*, as a decrease in brain mass is often seen in vLINCL patients. (**A**) Brain mass was reduced beginning at 5 months when compared to age matched controls. (**B**) Hippocampal and cerebral cortex volume were further assessed revealing a decrease in cortical volume at 9 months in the *Cln6^nclf^* mouse. (**C**) No difference in mean body weight is seen between adult WT and *Cln6^nclf^* mice up to 12 months of age. [Mean +/− SEM, *n* = 3 (**p≤0.01, ***p≤0.0001) for brain mass (A) and cortical volume measurements; *n* = 7–12 for body weight measurements]].

**Figure 4:**
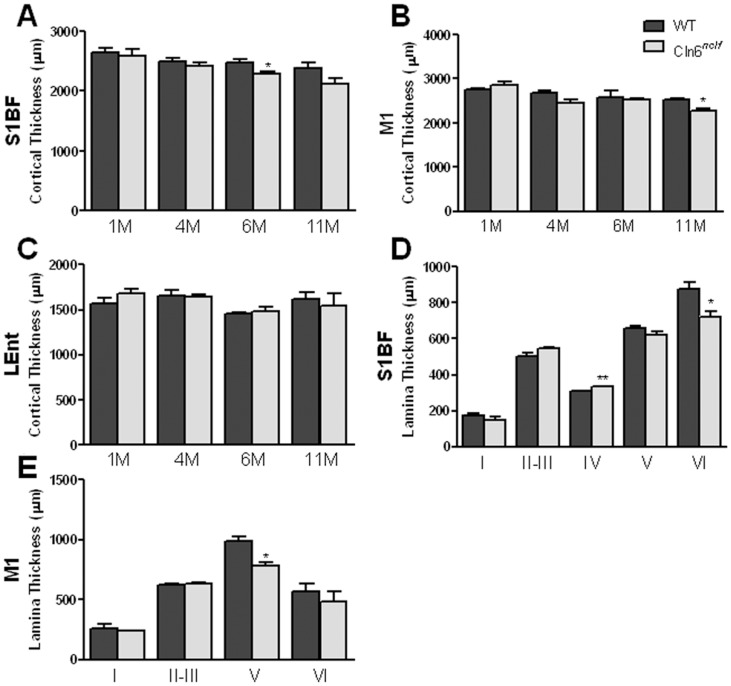
Cortical atrophy limited in *Cln6^nclf^* mice. Cortical thickness was evaluated in age matched WT and *Cln6^nclf^* mice. (**A**) In the S1BF, thinning begins to appear at 6 months. (**B**) Thinning in the M1 region is seen at 11 months. (**C**) Cortical thickness was unchanged in the LEnt (**C**) region of the cerebral cortex. (D–E) The S1BF (**D**) and M1 (**E**) regions were analyzed for possible laminar specific atrophy. Thinning is seen in lamina V of the M1 region at 11 months as well as an apparent thickening in lamina IV. Thinning is seen in the S1BF region in lamina VI at 6 months. [Mean +/− SEM (*n* = 3,*p≤0.05, **p≤0.01)].

Cortical atrophy, specifically in the primary motor (M1) and somatosensory barrel field (S1BF) cortex, has been widely reported in NCL animal models [26], [47], [52], [57], [58]. Therefore, we next examined *Cln6^nclf^* mice for signs of cortical atrophy and observed a significant loss of brain mass at 5 months and further decreased by 9 months, while the overall weight of the mice remained unchanged (Fig. 3 A, C). Atrophy was prominent in the cerebral cortex, with a reduction in volume seen in the neocortex at 9 months (Fig. 3 B). To discern which subregions of the cerebral cortex where affected, we measured thickness of the S1BF, M1, and lateral entorhinal (LEnt) (Fig. 4 A–C) cortex. Cortical atrophy appeared to be restricted to the S1BF and M1 regions, with an 8% reduction in S1BF cortical thickness at 6 months and a 10% reduction in M1 by 11 months (Fig. 4 A–B).

Neurons within individual cortical laminae are morphologically unique, projecting axons to specific regions within the CNS and, thus, cell loss within specific lamina can produce distinct phenotypes. In *Cln6^nclf^* mutant mice, lamina V of the M1 was decreased by 21% whereas lamina VI within the S1BF was reduced by 17% (Fig. 4 D–E) while lamina IV (in S1BF) increased in thickness (Fig. 4 E). These findings demonstrate a selective disruption within the medial portions of the cerebral cortex, including the M1 and S1BF, similar to what has been observed in patients with NCLs and other NCL models [26], [45], [47], [52], [57], [58], [59]. This cortical atrophy moved us to next examine other potential pathological changes that may be occurring within the cerebral cortex.

**Figure 5:**
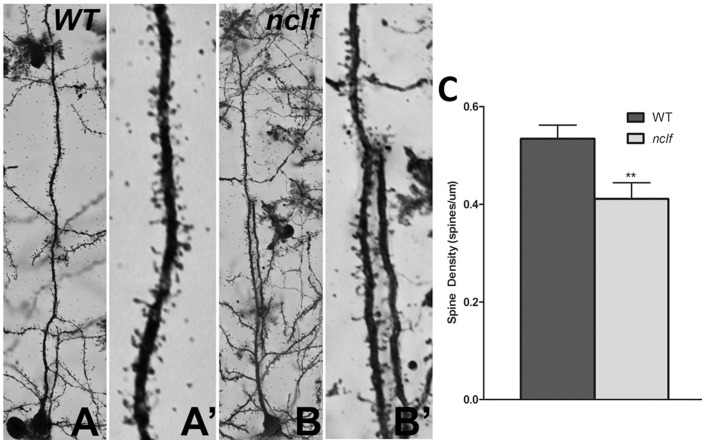
Decreased dendritic spine density in the mature cortex of *Cln6^nclf^* mice. Golgi impregnation was used to label dendritic spines on the primary dendrite of excitatory cortical projection neurons in age-matched 2 month controls (**A**) and *Cln6^nclf^* (**B**) mice. **(C)** The *Cln6^nclf^* mutant mouse excitatory pyramidal neurons have reduced spine density. [Mean spine density +/− SEM, *n* = 40 neurons counted per genotype (***p≤0.0001)].

Deficits in dendritic spine morphology have been implicated in the pathogenesis of numerous neurological disorders (Reviewed in [60]). Cortical pyramidal neurons have a single apical dendrite that extends towards the pial surface of the neocortex before undergoing extensive branching. Dendrites contain thousands of spines, sites of post-synaptic connection with other excitatory neurons and inhibitory interneurons. These spines are relatively plastic in normal adults. However, as age and neurologic disease set in, they become vulnerable to morphological changes and instability that may contribute to memory loss and/or motor deficits, depending on the region of the brain affected. Previous NCL animal model studies have revealed defects in synapse formation and stability [61] as well as a reduction in synaptic proteins specifically in *Cln6^nclf^* mice [30], [31], therefore we examined the dendritic spine morphology in the synaptic deficient M1 and S1BF regions of the *Cln6^nclf^* cortex. Using Golgi-Cox impregnation to examine dendritic spines, we observed a reduction in spine density on primary apical dendrites of pyramidal neurons of layers III–VI of the medial cerebral cortex in *Cln6^nclf^* mice (Fig. 5 A–C). Further analysis of spine head area and spine length as well as populations of size ranges revealed no differences in the morphology of the spines (spine head area p = 0.0748, spine length p = 0.9985). These results indicate that within the medial cortex of *Cln6^nclf^* mice, a region encompassing both the motor/somatosensory cortex, there is a defect in dendritic spine and synapse formation of the glutamatergic excitatory pyramidal neurons.

**Figure 6:**
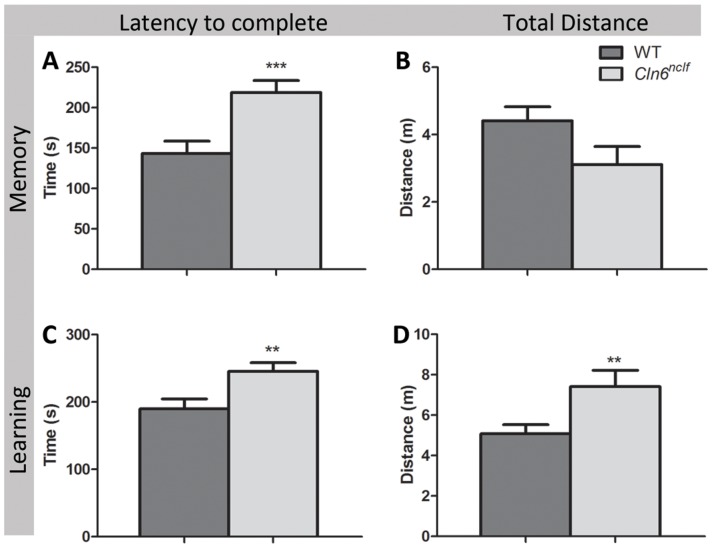
Learning and memory deficits are associated with mutation in *Cln6*. Following a period of habituation and training, memory and learning performance were tested in a radial arm maze task. **(A–B)** In an assay of memory, *Cln6^nclf^* mice displayed a significant increase in the latency (time in seconds) to complete an 8-arm radial maze task during a 5-trial session. **(C–D)** Deficits in the mean amount of time required to complete the task **(C)** and the mean total distance traveled (in meters) to complete the correct maze sequence **(D)** occurred in *Cln6^nclf^mice* when the maze task was altered 24-hours later to assay learning. [Mean +/− SEM, *n* = 6–9 (**p≤0.01, ***p≤0.0001)].

In humans, cortical atrophy and diminished synaptic activity often correlates with diminished cognitive function. To assay learning and memory performance, we performed an 8-arm radial arm maze on 8 month old mice. Mice were habituated and trained with a set sequence of rewarded arms for 20 days. Memory was assessed by recording latency, total distance traveled, and number of errors in completing the assigned test sequence. On the subsequent day the sequence of rewarded arms was changed and the latency, distance traveled and numbers of errors to complete the sequence was measured as an assay for learning [43]. *Cln6^nclf^* mice required a significantly longer amount of time to complete the test sequence of memory performance (Fig. 6 A). Moreover, when the testing sequence was altered to measure learning, *Cln6^nclf^* mice had a longer latency to complete the task and traveled a greater distance (Fig. 6 C–D). These parameters demonstrate that mutations in *Cln6^nclf^* impair both learning and memory, mimicking the cognitive deficits seen in human NCL patients.

### Astrocytic and microglial activation specific to subregions of the cerebral cortex

**Figure 7:**
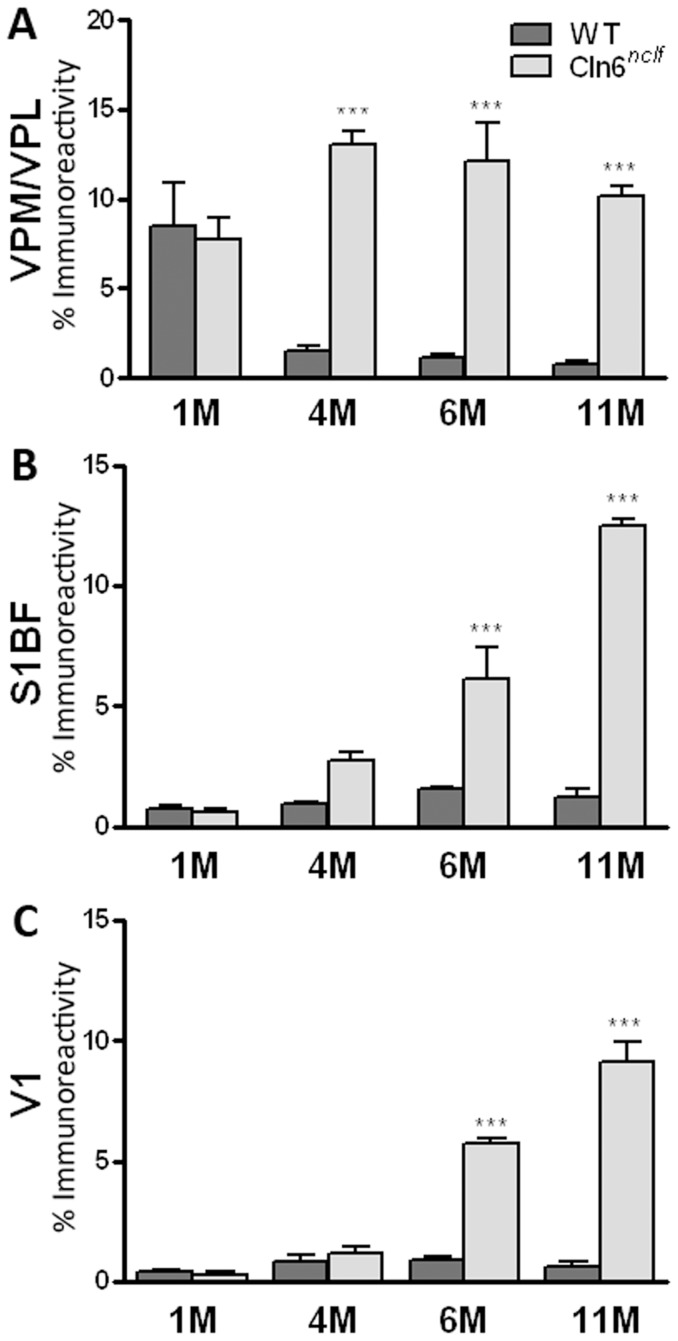
Upregulation of astrocyte marker GFAP in the thalamus and cerebral cortex of *Cln6^nclf^* mice. (**A**) Quantitative thresholding analysis revealed a significant increase in the expression of astrocytic markers in the *Cln6^nclf^* mouse at 4 months in the thalamus. (**B–C**) Elevations in astrocytic activation, marked by GFAP labeling, became apparent in the S1BF region at 4 months and in the V1 region of the cerebral cortex at 6 months in the *Cln6^nclf^* mouse. [Mean% immunoreactivity +/− SEM, *n* = 3 (*p≤0.05, **p≤0.01, ***p≤0.0001)].

**Figure 8:**
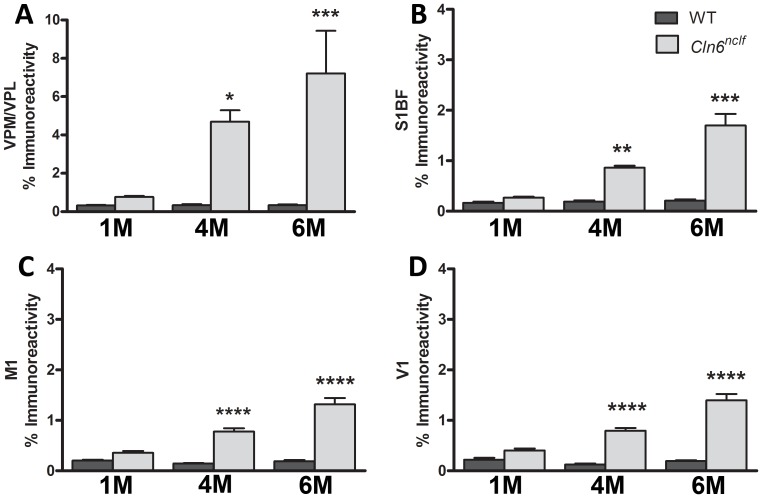
Upregulation of microglial marker CD68 in the thalamus and cerebral cortex of *Cln6^nclf^* mice. Quantitative thresholding analysis in *Cln6^nclf^* mice was compared to age matched WT, revealing a significant increase in the expression of the microglia marker CD68 in the VPM/VPL (**A**), M1 (**C**), S1BF (**B**), and V1 (**D**) regions in the *Cln6^nclf^* mouse over WT. [Mean% immunoreactivity +/− SEM, *n* = 3 (*p≤0.05, **p≤0.01, ***p≤0.001, ****p≤0.0001)].

Another pathological hallmark of the NCLs is an early onset, robust activation of glia activation. To quantify the activation of astrocytes and microglia, threshold analyses of GFAP (glial fibrillary acidic protein, astrocytes) and CD68 (microglia) immunoreactivity were performed. Levels of GFAP staining were measured in the S1BF and V1 regions of the cerebral cortex and the medial and lateral parts of the ventroposterior nucleus of the thalamus (VPM/VPL) (Fig. 7 A–C), another brain region often affected early in NCL mouse models [47], [50], [52], [57], [58], [62], [63]. Within the S1BF and V1 regions, there was a marked increase in GFAP by 6 months of age (Fig. 7 B–C). When analyzing VPM/VPL thalamic nuclei, GFAP immunoreactivity was massively upregulated at 4 months, persisting with age (Fig. 7 A). Microglial activation was apparent in all of the regions examined, appearing by 4 months of age and remaining elevated throughout the life of the animal [VPM/VPL, S1BF, M1, and V1, (Fig. 8 A–D)]. Although glial activation has been reported previously in *Cln6^nclf^* mice, these findings demonstrate earlier onset pathology, with previous studies showing astrocytosis at 21 weeks and microglial activation at 54 weeks of age [32].

### Interneuron loss within the mature *Cln6^nclf^* cortex and hippocampus

**Figure 9:**
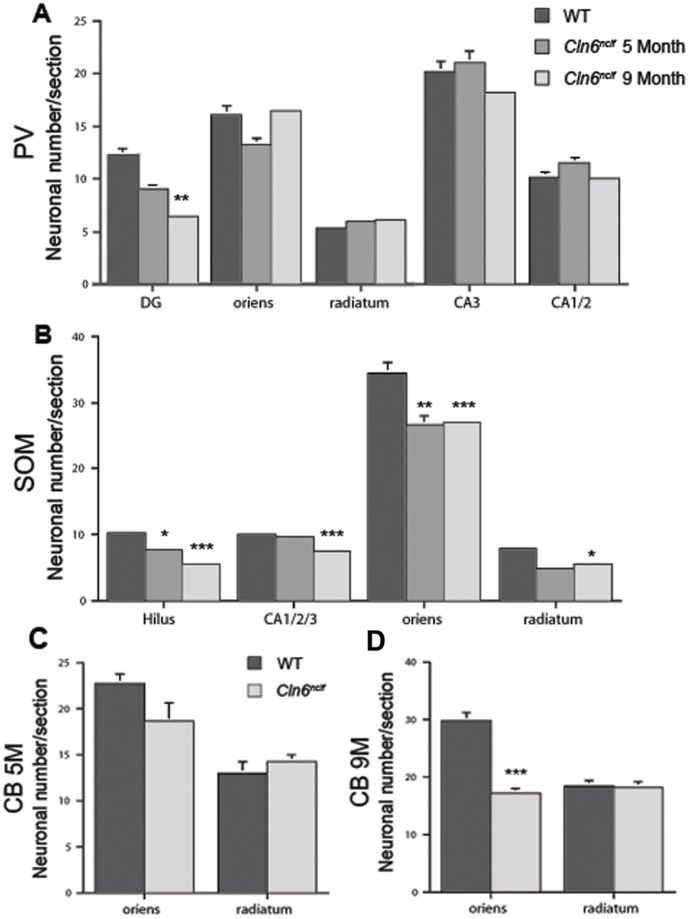
Loss of specific subpopulations of interneurons seen within various regions of the hippocampus. Loss of interneurons is a characteristic often seen in the hippocampus of the NCLs. We divided the hippocampus into defined regions and stained sections for specific subpopulations of interneurons and subsequently counted the subpopulations within the sections. (**A**) PV+ interneurons decreased in the dentate gyrus (DG) at 9 months but remain unchanged in other areas where PV+ interneurons were present. (**B**) SOM+ interneurons were first decreased at 5 months in the hilus and stratum oriens with further decline observed at 9 months. Decline of SOM+ interneurons was also seen at 9 months in the stratum radiatum as well as the combined CA fields of the hippocampus. (**C–D**) A significant reduction in the number of CB+ interneurons was observed within the stratum oriens at 9 months when compared to WT. [Mean +/− SEM, *n* = 3 (*p≤0.05, **p≤0.01, ***p≤0.001, ****p≤0.0001)].

Loss of γ-amino butyric acid (GABA) inhibitory cortical neurons within the cerebral cortex and hippocampus has been observed in human NCLs patients [64], [65], and NCL animal models [18], [45], [47], [48], [50], [59], [66]. Studies of CLN6 sheep models have demonstrated loss in distinct subtypes of interneurons, varying within the cortical subregion examined [59]. Specifically, parvalbumin (PV) and somatostatin (SOM) positive interneurons in the parietal cortex appear selectively vulnerable whereas calretinin (CR) and calbindin (CB) positive interneurons remain relatively unaffected [59]. Here, we reveal a similar phenomenon in *Cln6^nclf^* mice. SOM+, PV+, and CB+ interneurons all exhibit some deficiency within the *Cln6^nclf^* brain (Fig. 9 and Fig. S2). Within the LEnt cortex, a region identified as having fewer GABAergic interneurons in other NCL models [45],[59], there was a decline in PV+ interneurons (Fig. S2). Interneuron loss within the hippocampus varied across subfields. In the dentate gyrus, there was a slight decline in PV+ interneurons at 5 months, with increasing severity at 9 months (Fig. 9 A). Beginning at 5 months, SOM+ interneuron loss was seen in the dentate gyrus/hilus and stratum oriens, with loss extending to the stratum radiatum and combined CA fields 1, 2, and 3 at 9 months (Fig. 9 B). CB+ interneurons within the *Cln6^nclf^* hippocampus were markedly reduced only at the later timepoint (Fig. 9 C–D), with this cell loss restricted to the stratum oriens. These findings demonstrate significant reduction in select sub-populations of hippocampal interneurons in *Cln6^nclf^* mice, similar to other NCL animals and NCL patients.

## Discussion

The NCLs are a devastating family of genetic disorders which manifests in early childhood with vision loss, motor decline, seizures, and culminates in premature death (Reviewed in [7]). The ability to recapitulate these diseases in animal models provides invaluable tools for defining the underlying molecular mechanisms responsible for the NCLs and for successful screening of potential therapies. Spontaneous mutations in *Cln6* have arisen in several different species resulting in a murine (*Cln6^nclf^*) model [21], two ovine models [20], [67], [68], and a canine model [22]. Here, we dissect the pathology and behavioral phenotype of *Cln6^nclf^* mutant mice and demonstrate that these mice closely recapitulate the disease progression seen in human patients, elevating their validity for therapeutic drug screening studies.

To date, 55 disease-causing mutations have been identified in *CLN6* (see http://www.ucl.ac.uk/ncl/cln6.shtml). Recent studies have also linked mutations in CLN6 to the adult-onset form of NCL (or Kufs disease, Type A) [9]. In both vLINCL and Kufs A, mutations span the entire gene yet offer no indication of a relation between the mutations and phenotype. The mutation in *Cln6^nclf^* mice is homologous to a mutation in a Pakistani family with vLINCL [11], [13]. The human and murine CLN6 show 90.3% amino acid similarity [11], with *Cln6^nclf^* mutation (c.307insC, frameshift after P102) located on exon 4 [11], [13]. This insertion results in a frameshift followed by 25 novel amino acids and a premature stop codon [11]. Human mutations in *CLN6* have been found in seven different exons, all resulting in similar pathological features [16] – providing no pattern in severity of disease symptoms and age of onset making it difficult to pinpoint domains that are critical for proper protein function [8], [10], [13], [15], [16], [69]. Access to these reliable, well validated animal models of vLINCL will allow scientists to better understand both the genotype/phenotype relationship of these mutations.

One common feature for many of the NCLs is retinal deterioration and eventual blindness. This hallmark degeneration of the retina has been seen in other NCL animal models [44], [70], [71], [72], [73], [74], [75], [76]. Here we show that cell loss in the *Cln6^nclf^* retina begins around 3 months of age and progresses rapidly. The exact cause of retinal degeneration and eventual blindness in the NCLs is unclear and likely varies with subtype of the disease. A variety of hypotheses have been proposed such as altered gene expression, accumulation of storage bodies resulting in deficient phagocytosis, as well as possible degeneration in the optic nerve, lateral geniculate and/or occipital lobe resulting in progressive retinal degradation [44], [77], [78]. Furthermore, not all NCL animal models exhibit the same degree of retinal degeneration and visual loss, indicating that some compensation may be made at different levels, with convergent pathways that eventually result in neuronal loss [76]. This is further evident in NCL patients, since not all patients display the same levels of degeneration [8], [10], [13], [15], [16], [69].

vLINCL patients also exhibit motor delay, dystharthia, ataxia and eventual paralysis. Here, we are able to recapitulate motor deficits in *Cln6^nclf^* mice using several tests of balance and coordination. By as early as 3 months, diminished motor coordination could be observed. Previously, Bronson et al. have reported rear-limb paralysis starting at 10 months of age [21] with more recent studies demonstrating rotarod motor coordination deficits around the same time (at ∼45 weeks of age [33]). Here we provided an earlier behavioral indicator of disease progression, enhancing the utility of this model for therapeutic/drug screening. One should be cautioned though that a recent study of another NCL mouse model has shown that changes in the animal's environment can significantly impact their motor performance (including the age of onset of deficits) – thus changes in housing or diet may contribute to the manifestation of these earlier behavioral phenotypes [79].

In both vLINCL human patients and the CLN6 sheep models there is progressive thinning of the cerebral cortex and neuronal loss ([16], [18], [59]; reviewed in [80]). We show here that these changes are recapitulated in *Cln6^nclf^* mice, with a reduction in brain mass and thinning in the S1BF and M1 cortex by 9 months of age. Additionally, we report here a selective loss of GABAergic inhibitory interneurons in the cortex and hippocampus, similar to what has been reported in vLINCL patients and other NCL animal models [18], [45], [48], [59], [66]. Specifically, we demonstrate that within *Cln6^nclf^* cortices there is a specific loss of PV+ interneurons within the LEnt cortex, and further loss of PV+, SOM+, and CB+ interneurons in subdomains of the hippocampus, mirroring what has been reported in the South Hampshire sheep model [59]. Further research into the cause of interneuron loss as well as the timing of cell loss will be needed to understand the role this plays in disease pathology.

Dysregulated synapse morphology has been implicated in a number of neurodegenerative and neurological diseases [81]. Indeed, loss of dendrite spines and synaptic connectivity has been associated with a host of diseases including epilepsy, schizophrenia, autism, fragile X, mental retardation, and Alzheimer's disease [82], [83], [84], [85], [86], [87], [88]. The reduction in dendritic spine density reported here may contribute to the decline in motor performance, learning and memory observed in *Cln6^nclf^* mice model and may be directly related to previously reported disruption in synaptic and axonal protein levels [31]. Combined, these findings of early onset disruption in dendrite spine density might suggest earlier dysfunction within the cortex that precedes neuronal loss.

Glia play an essential role in neuronal function, including the regulation of spine pathology and synapse formation [89]. Elevated astrocytosis and microglial activation has been reported in sheep models of NCLs – starting as early as 20 days *before* birth with increasing activation through maturation [26], [90], [91], [92]. In *Cln6^nclf^* mice however, the cortical astrocytosis does not appear until later at 6 months. Microglial activation however mirrors the sheep model, appearing early in life (by 1 month of age). Further investigation of prenatal and early postnatal time points will allow us to narrow down a more precise window of activation.

Taken together our findings provide novel insights into the pathogenesis of this disease, and based upon the evidence provided, the onset of this disease may be occurring earlier than previously reported. We demonstrate novel defects in visual acuity, learning, and memory as well as pathological deficits in dendrite spine morphology. Combined, these data establish *Cln6^nclf^* mice as an essential tool for the study of vLINCL, presenting phenotypic similarities to NCL patients and other NCL animal models. The *Cln6^nclf^* mouse model will therefore provide an invaluable platform for high throughput screening of possible NCL therapeutics.

## Supporting Information

Figure S1 **Accumulation of autofluorescent storage material in the** ***Cln6^nclf^*** **mouse.** Confocal microscopy images of age matched WT and *Cln6^nclf^* mutant mice were taken to assess the accumulation autofluorescent storage material of cortical sections. The accumulation of storage material becomes apparent in the mutant cortex by 5 months (upper right panel) of age and increases through 9 months (lower right panel) with no accumulation seen in the WT cortex (left panels).
(TIF)

Figure S2 ***Cln6^nclf^*** **mice exhibit a loss of parvalbumin interneurons in specific subregions of the cerebral cortex.** Interneuron loss is often seen in NCL patients as well as animal models. Interneuron subpopulations were stained and counted within the entorhinal region of the cerebral cortex which resulted in an observed decrease in PV+ interneurons within the cortex versus age matched controls. [Mean +/− SEM, *n* = 3 (**p≤0.01)]
(TIF)
