# Supplementary figures and images for: A Murine Model of Variant Late Infantile Ceroid Lipofuscinosis Recapitulates Behavioral and Pathological Phenotypes of Human Disease
Source: PLoS One. 2013 Nov 1;8(11):e78694. doi: 10.1371/journal.pone.0078694 (PMC3815212; doi:10.1371/journal.pone.0078694)

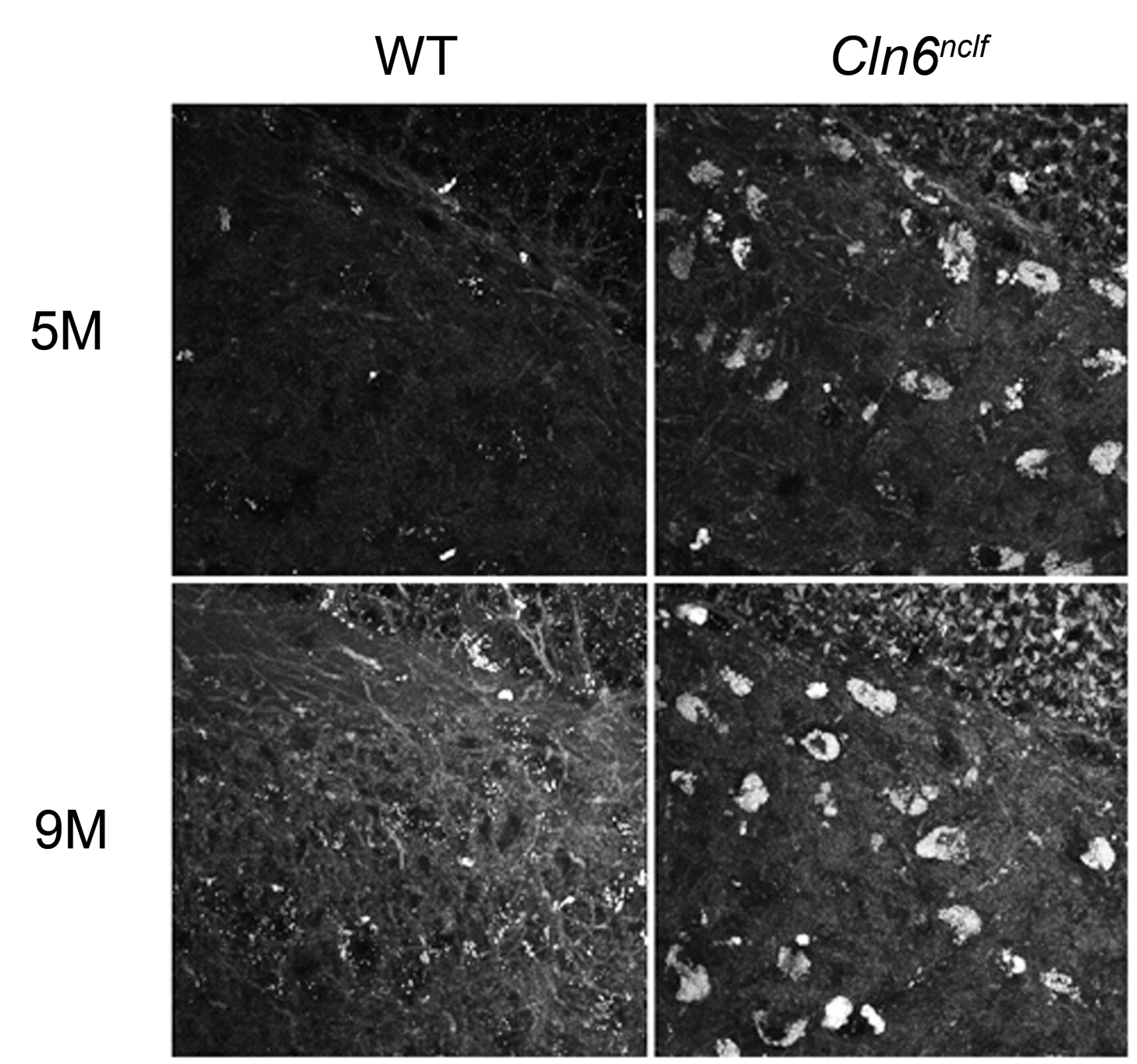

Supplement: Figure S1 — Accumulation of autofluorescent storage material in the Cln6nclf mouse. Confocal microscopy images of age matched WT and Cln6nclf mutant mice were taken to assess the accumulation autofluorescent storage material of cortical sections. The accumulation of storage material becomes apparent in the mutant cortex by 5 months (upper right panel) of age and increases through 9 months (lower right panel) with no accumulation seen in the WT cortex (left panels). (TIF) [file pone.0078694.s001.tif]

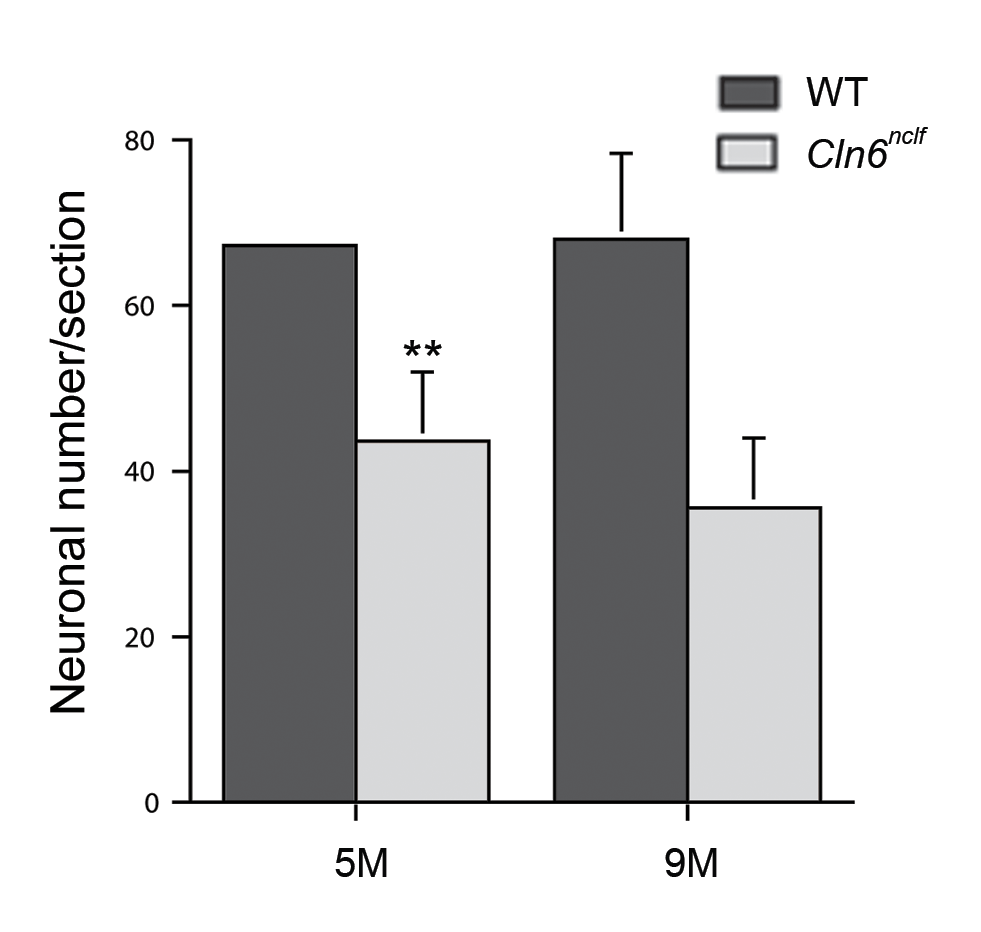

Supplement: Figure S2 — Cln6nclf mice exhibit a loss of parvalbumin interneurons in specific subregions of the cerebral cortex. Interneuron loss is often seen in NCL patients as well as animal models. Interneuron subpopulations were stained and counted within the entorhinal region of the cerebral cortex which resulted in an observed decrease in PV+ interneurons within the cortex versus age matched controls. [Mean +/− SEM, n = 3 (**p≤0.01)] (TIF) [file pone.0078694.s002.tif]
